# American Medical Association—Eleventh Annual Meeting

**Published:** 1858-06

**Authors:** 


### *American Medical Association—Eleventh Annual Meeting.*

*First Day*—The Association met in the lecture-room of the Smithsonian Institution, and was called to order at a quarter past 11 o'clock, A. M., by Dr. Condie, of Philadelphia, when the Chair was taken by the President, Dr. Paul F. Eve, of Nashville, Tennessee. Vice-Presidents Breckinridge, of Kentucky, Reese, of New York, and Campbell, of Georgia, were also on the platform; and at their tables were the efficient Secretaries, Drs. Foster, of Tennessee, and Semmes, of Washington.

Rev. Byron Sunderland, D. D., at the invitation of the President, offered an eloquent and appropriate prayer, invoking the blessings of Almighty God upon the Convention.

Dr. Harvey Lindsley, chairman of the Committee of Arrangements, then delivered an address of welcome.

The Secretary then called the roll by States, as it had been made out up to the commencement of the meeting, and the following number of delegates responded:

Maine 2, New Hampshire 8, Connecticut 18, Vermont 1, Massachusetts 40, Rhode Island 5, New York 73, New Jersey 25, Pennsylvania 66, Delaware 4, Maryland 24, District of Columbia 25, Virginia 8, North Carolina 8, South Carolina 10, Georgia 12, Alabama 1, Kentucky 9, Tennessee 7, Indiana 6, Illinois 12, Michigan 3, Iowa 3, Missouri 4, Ohio 14, California 1, American Medical Society of Paris 1, U. S. Navy 2.

Dr. Lindsley, chairmnn of the Committee of Arrangements, reported that it had been decided to hold but one business session each day, from 9 A. M., until 3 P. M. He also announced that the President of the United States would be happy to receive, with those members of the Association who might call at the Executive Mansion at 8 o'clock in the evening, such ladies as may accompany them.

On motion, the Association confirmed the appointment of Dr. J. M. Snyder to fill a vacancy in the Committee of Arrangements.

On motion, it was decided that a nominating committee of one from each State represented, should be raised, the delegation of each State selecting its representative therein. A brief discussion upon the propriety of permitting the Army and Navy delegations to appoint separate members of the committee was decided by the President in favor of their having the privilege, and the decision was sustained by the Association.

There was then a recess of fifteen minutes, during which the different delegations assembled in various parts of the lecture-room to choose their representatives in the committee. After the meeting was again called to order, the Secretary read the list as follows:

*Committee of Nomination.*—Job Holmes, of Maine; Geo. H. Hubbard, New Hampshire; P. Pineo, Vermont; Ebenezer Alden, Massachusetts; Ashbel Woodward, Connecticut; J. Mauran, Rhode Island; H. D. Bulkley, New York; J. P. Colman, New Jersey; Isaac Hays, Pennsylvania; H. F. Askew, Delaware; P. Smith, Maryland; Noble Young, District of Columbia; A. S. Payne, Virginia; W. H. McKee, North Carolina; Wm. T. Wragg, South Carolina; Joseph P. Logan, Georgia; J. T. Hargraves, Alabama; R. J. Breckinridge, Kentucky; J. Berrien

Lindsley, Tennessee; Wm. M. McPheeters, Missouri; George Mendenhall, Ohio; Calvin West, Indiana; A. H. Luce, Illinois; Zina Pitcher, Michigan; Thomas O. Edwards, Iowa; O. Harvey, California, and Geo. Clymer, United States Navy.

On motion, Drs. Bohrer, of D. C., Flint, of New York, and Hargraves, of Alabama, were appointed by the President a Committee on Special Essays.

Dr. David M. Reese, of New York, presented and read a written apology for having recommended for a position in Blockley Hospital, Philadelphia, Dr. McClintock, who had been expelled from the Association for a violation of the ethics and the etiquette of the profession, by publishing a work on "domestic medicine," &c.

On motion of Dr. Condie, of Philadelphia, the apology was accepted, and ordered to be entered upon the minutes.

Dr. Bryan, of Philadelphia, who had also recommended Dr. McClintock, made a verbal adoption of Dr. Reese's apology, the reception of which was warmly debated. Dr. C. C. Cox, of Maryland, opposed, and Dr. Condie advocated the reception. Dr. A. B. Palmer, of Michigan, moved the previous question on a motion to refer the subject to a committee, which was lost. The apology of Dr. Bryan was then accepted.

The President then delivered, in a clear voice, and with a pleasing oratorical effect, his Annual Address.

On motion, the thanks of the Association were voted to the President for his able and instructive address, a copy of which was solicited for publication.

Dr. Grafton Tyler, of Georgetown, D. C., chairman of the Committee on Prize Essays, reported that the essays received were three in number, each of which had been examined with great care; considering, first, the intrinsic merits of each essay, and then their merits in relation to each other. The first prize was awarded to "An Essay on the Clinical Study of the Heart Sounds, in Health and Disease," bearing the motto: "*Clinica clinice Demonstrandum.*" The second prize was awarded to "An Essay on Vision and some of the Anomalies as rendered by the Opthalmoscope," bearing the motto: "*Dux hominum medicus est.*"

Dr. Tyler then proceeded to open the sealed envelopes bearing the above named mottoes, and containing the names of the writers of the Essays. The first was written by Dr. Austin Flint, of Buffalo, N. Y.; and the second by Dr. Montrose A. Pallen, of St. Louis, Mo. This is the second time Dr. Flint has won this distinguished honor, and the third time that it has been awarded to Buffalo since the Association was organized, eleven years ago.

On motion, the report of the Committee was accepted and adopted. Drs. Flint and Pallen were then invited to give *resumes* of their essays, which they did.

Dr. Lindsley, from the Committee of Arrangements, then presented an invitation from Dr. Nichols to visit the Insane Asylum, and another from Rev. Mr. McGuire, to visit Georgetown College.

On motion of Dr. Hamilton, of New York, these invitations were accepted, and the thanks of the Association were returned therefor.

On motion of Dr. Lindsley, the Hon. Doctors Fitch, of Indiana, Chaffee, of Massachusetts, Clawson and Robbins, of New Jersey, and Shaw, of North Carolina, members of Congress, and Dr. Peter Parker, ex-commissioner to China, were elected "members by invitation," and requested to participate in the proceedings of the Association.

On motion, Assistant Surgeon Frederick A. Rose, of the British Navy, who so nobly volunteered his services on board the United States ship Susquehanna, at Port Royal, and who came in her to New York, devoting himself to the sick crew, was unanimously elected a "member by invitation," and invited to take a seat cn the platform. It was announced that Dr. Rose had left the city.

Dr Francis G. Smith, of Philadelphia, chairman of the Committee on Publication, made his report, showing the expense of publishing the annual volume.

Dr. Caspar Wistar, of Philadelphia, presented his annual report of receipts and expenditures, showing a balance on hand of $806. Accompanying the Treasurer's report was a resolution providing that the back volumes on hand, when over two years old, shall be sold at two dollars a volume, and that vols. V, VII, VIII and IX, of which there are a surplus, be sold at $5 a set to any member.

The special committee on Medical Education, of which Dr. G. W. Norris, of Philadelphia, is chairman, were called upon to report. There was no response, and, on motion, the subject was referred to the Committee on Nominations.

Dr. A. B. Palmer, chairman of the Committee on Medical Literature, asked leave to defer his report until Wednesday, at 10 o'clock, which was granted.

A report was made by the Committee on Nominations; which was accepted; and the Association then elected the following Officers:

President, Dr. Harvey Lindsley, of Washington City. Vice-Presidents, Drs. W. L. Sutton, of Kentucky; Thos. O. Edwards, of Iowa; Josiah Crosby, of New Hampshire; and W. C. Warren, of North Carolina. Secretary, Dr. A. J. Semmes, of Washington City. Treasurer, Caspar Wistar, of Philadelphia.

On motion, Drs. Flint, of New York. Gross, of Pennsylvania, and Gibbes, of South Carolina, were appointed a committee to conduct the President elect to the Chair.

Dr. Lindsley, having been introduced to the Association by the retiring President, Dr. Eve, made a few pertinent remarks acknowledging the honor as the highest he had ever been called upon to receive, and the highest that any medical man in America can receive. Unaccustomed to preside over so large a body, and having had but little practice in presiding over smaller assemblages, he must throw himself upon the forbearance of the association, and look to the members for support in the discharge of his official duties.

On motion, the thanks of the Association were voted to the retiring officers for the able and impartial manner in which they have discharged the duties of their respective offices.

On motion, the ex-Presidents of the Association present were invited to take seats on the platform.

The committee on Medical Topography and Epidemics was called by States. A paper from the member from Maine stated that he will report next year. There was no response from New Hampshire, Vermont, Rhode Island, Connecticut or Massachusetts. Dr. Smith, of New Jersey, read an able report on New Jersey, and the Association then adjourned until to-morrow morning at 9 o'clock.

*Second Day*—The Association was called to order by the President, Dr. Harvey Lindsley, and the Secretary read the minutes of the first day's proceedings, which were adopted.

On motion of Dr. Watson, of New York, Dr. Delafield, of New York, one of the first officers of the association, was invited to take a seat on the platform.

On motion of Dr. Atkinson, of Virginia, an amendment to the constitution was received, providing that no person shall be recognized as a member, or admitted as a delegate at meetings of the association who has been expelled from any State or local medical association until relieved by action of such State or local association.

Dr. Atkinson supported the adoption of this amendment in an eloquent speech, contending that the admission of any gentleman who has been rebuked by any State or local association, and is under its ban, is a rebuke to that association. He urged the acceptance of the amendment, and trusted that until the constitution be so amended it shall be the rule of action.

Dr. Bond, of Maryland, asked to have the qualifications requisite for a seat read. He desired information as to the ethical qualifications for membership.

Dr. Watson, of New York, stated that, as by the constitution it was necessary to have amendments lie over one year, this was not a question for present debate.

The President decided that debate was not in order, and the amendment was accordingly laid on· the table for consideration at the next annuel meeting.

Dr. Boyle, of the committee of arrangments, proposed the names of Doctors Huff and Night, who were elected "members by invitation.

Dr. A. B. Palmer, of Michigan, chairman of the committee on medical literature, made an able and interesting report.

On motion, the report was accepted, and ordered to be published.

On motion, Dr. Bozeman, of Alabama, was elected a "member by invitation.

REPORT ON MEDICAL EDUCATION.

Dr. James R. Wood, chairman of a special committe on medical education, made a lengthy report, discussing; 1st, primary medical schools; 2d, the number of professorships in medical colleges; 3d, the length and number of terms during the year; 4th, the requisite qualifications for graduation; 5th, such other subjects of a general character as to give uniformity to our medical system. Having reviewed these propostions at length, the committee have arrived at the following conclusions.

*First* Primary medical shools should be encouraged; but, as office i n instruction will continue to be sought by students, practitioners should either give them necessary advantages of demonstration, illustrations, and recitations, or if not prepared to do so, they should refer them to such primary shools, or medical men, as will give them proper insruction.

*Second.* The number of professorships should not be less than seven, viz.: a Professor of Anatomy and Microscopy, Physiology and Pathology, Chemistry, Surgery, Practical Medicine, Obstetrics, and Materia Medica.

*Third.* There should be but one term annually which should commence about the 1st of October, and close with the March following, thus lengthening the term to six months. The commencement of the term, in October, should be uniform in all the colleges throughout the country. During the session their should never be more than four lec. tures given daily.

*Fourth.* The qualifications for graduation, in addition to those now required by the schools, should be a liberal primary education, and attendance upon a course of clinical instruction in a regularly-organized hospital.

In order to give our medical colleges an opportunity to consider the recommendations here advanced, and that this body may have the advantage of their wisdom and their mature views, before any definite action is taken upon them, your committee submit to the association the following resolutions:

*Resolved,* That the several medical colleges of the Unitad States be requested to send delegates to a convention to be held at on the day of for the purpose of devising a uniform system of medical educaiton.

*Resolved,* That the present report of the special committee on medical education be referred to such convention for its consideration.

*Resolved,* That said convention of delegates from the several colleges of the United States be requested to submit to the meeting of this association in May 1859, the result of their deliberations.

On motion, the report was accepted and referred to the committee on publication, the accompanying resolution be laid on the table.

The committee on nominations reported Louisville, Ky., as the place of meeting in 1859, and nominated Dr. S. M. Bemis, of that city. as second Secretary. They also nominated the standing committees.

*Committee on Publication*—Dr. F. Gurney Smith, Pa., Chairman; Drs. Caspar Wistar, Pa., A. J. Semmes, D. C., S. M. Bemis, Ky., S. L. Hollingsworth, Pa., S. Lewis, Pa., H. F. Askew, Del.

*Committee on Medical Literature*—Dr. John Watson, N. Y., chairman; Drs. L. A. Smith, N. J., C. G. Comegys Ohio, R. W. Gibbs, S. C., W. M. McPheeters, Mo.

*Committee on Prize Essays*—Dr. J. B. Flint, Ky., chairman; Drs. M Goldsmith. N. J., H. Miller, Ky., Calvin West Ind.

*Committee on Medical Education*—Dr. G. W. Norris, Pa., chairman; Drs. A. H. Luse, Ill., E. Henderson, S. C., G. R. Grant, Tenn., T. S. Powell, Ga.

*Committee of Arrangments*—R. J. Breckinridge, Ky., chairman; Drs. G. W. Ronald, B. M. Wible, D. W. Goodall, D. D. Thompson, N. B. Marihall, G. W. Burglass, R. C. Hewett and A. B. Cook, all of Kentucky.

The report was accepted, the nominations were confirmed, and the committee received permission to sit again.

On motion of Dr. Hamiltion, of Baffalo, the resolutions attached to the report of the committee on medical education were taken from the table.

Dr. Watson moved the appointment of a committee to consider the resolutions and report to-morrow morning.

Dr. Bond thought that the subject had already been sufficiently discussed. It had been brought up year after year, occupying much of the time of the association, and he trusted that it would receive immediate consideration.

Dr. Davis, of Illinois, wished, to have the subject made a special order for some time prior to the adjournment of the convention.

Dr. Rogers, of New York, wished to have the report printed, that all might have an opportunity of examining it and the propositions which it embodies.

Dr. Wood defended his report as a conservative report, just alike to the profession and to the laymen. He did not believe that any good could arise from a further discussion of the subject. None has arisen in years past—none could arise now. It was a bill of conciliation and of adjustment. Laymen of the profession merited censure for sending men to college not qualified for the profession, and colleges merited censure for sending men out not qualified to practice the healing art. He approved of the motion of Dr. Watson, that the report be submitted to a committee of delegates from colleges.

A debate on a call for the previous question on Watson's resolution then ensued, in which several gentlemen joined, each one apparently having a different idea of "parliamentary law," and neither of them displaying a very correct knowledge of the subject. It was remarked by an old member of the association that "parliamentary discussion must be a *local epidemic.*"

Tha report was finally referred to a select committee, to be composed of one member from each delegation representing a medical college or school.

On motion, thanks were voted to the late secretary, Dr. Foster and his succesor, Dr. Bemis, took his seat.

Dr. Hamm, of Pennsylvania, moved a suspension of the rules for the purpose of reconsidering the resolution of Dr. Condie, accepting the apology tendered by Dr. Bryan. The vote upon suspending the rules stood —ayes 111, noes 82. The President ruled that a two-thirds vote was necessary, and decided the question as lost.

An appeal was taken from the decision of the chair, and the decision was not sustained. A vote was then taken, and the resolution accepting the apology of Dr. Bryan was reconsidered by a vote of—year 142, nays 70.

An attempt was then made to connect the resolution with that accepting the apology of Dr. Reese, but it was decided that it would first, be necessary to dispose of the resolution reconsidered, and it was laid on the table.

A member from New Jersey hoped that the McClintock case would be brought fairly and squarely before the association, and that gentlemen would be made to "face the music." It was useless to cloak it, or attempt to dodge the responsibility.

Dr. Beck, of Indiana, moved an indefinite postponement of the whole subject.

Other gentlemen rose to speak, but the President decided that a motion to postpone was not debatable.

Dr. Jewell rose to a point of order, and protested against being "gagged." [The President here reversed his decision.] Dr. Jewell said that the action of the day previous was regretted, and that gentlemen had acted hastily. Many, who voted at first sight to accept the apologies, now regretted having done so.

Dr. Hamm, of Philadelphia, explained the action of the Philadelphia County Medical Society, and began to read a remonstrance from it, which he desired to incorporate into his speech.

Dr. Biddle objected to the reading of this remonstrance, as a violation of plighted faith.

It was here moved and decided that the association go into "committee of the whole," and Dr. Edwards, of Ohio was called to the chair

A member hoped that there would be no rules of order except what the chair would prescribe.

*The Chair.* "I will prescribe enough."

Another member inquired if it would be proper to discuss the remonstance? *The Chair.* "A gentleman who has the floor can discuss anything on the face of the earth.

The remonstrance was then read. It was a long document, giving a detailed account of the recommendation by Dr. Reese of Dr. McClintock for a position in Blockley Hospital, after the last-named gentleman had been guilty of selling quack nostrums, and had thus committed an offence against the ethics of the profession.

Dr. Humphries, of Indiana, moved that each member of the committee of the whole be restricted to five minutes, allowing Dr. Reese whatever time he wished to defend himself in.

Dr. Phelps showed that a ten-minutes rule was now in force. Dr. Cox moved, as an amendment, to make the time fifteen minutes; which amendment was lost, and the original motion of Dr. Humphries was then carried.

Dr. Reese then ascended the platform, and made a statement of his position from the commencement of the controversy. He considered his apology of the day previous a satisfactory one, but was willing to make it more so if it was objected to. He had not brought the subject before association; but had been given to understand that if he made the apology which he made, the remonstrance would not be offered. During his remarks there was a demand for the reading of the apology; which was read, as follows:

*To the Officers and Members of the American Medical Association:* The undersigned, one of the Vice Presidents of the American Medical Association, having, during the interval since our last annual meeting, certified to the professional fitness for the charge of the Blockley Hospital, at Philadelphia, of an individual who had been expelled from this body for a violation of our code of ethics, after consultation with the other officers, and yielding to the advice of other personal friends, desires to say to the association now assembled—

1st. That, in giving said certificate, he was prompted solely by motives of sympathy and humanity to a fallen brother, who had been a personal friend prior to his offence; and that he did not realize, acting under the impulse of the moment, that his individual act could be construed by the profession as indicating hostility to his brethren.

2d. That while his own mind is clear that the certificate contained only the truth, and that, under his peculiar relations to the party concerned, he could not withhold his certificate, of medical qualification, consistent with conscience and duty, yet he is ready to concede that he had no abstract right to relieve the party from the censure of the association until this body had restored him to his fellowship.

3d. That, so far from intending any disrespect to the association or to its act of discipline, the undersigned had publicly sustained and defended both. He therefore disclaims the inference from his certificate that he intended to recommend to a high professional office a man whom the association had excluded, and thereby nullify the action of this body.

And, finally, with these statements and disclaimers, the undersigned, while retaining his own opinion of the rectitude of his motives, and of his duty, under the peculiar circumstances of the case, is nevertheless prepared to defer to the judgment of those whom he knows to be his friends, that he erred in doing what he had no right to do, in view of his official position in the association, and is hence called upon to offer this explanation and apology to his brethren.

(Signed) DAVID M. REESE.

It was moved to refer the apology and remarks of Dr. Reese to a special committee of seven, to report to-morrow morning. Dr. Atlee, of Lancaster, and other gentlemen urged delay.

Dr. Payne, of Virginia, asked permission to relate an anecdote. He was reminded of two old Quakers, one of whom kept a store, while the other practised law—both were members of a temperance society, and it was generally thought that the lawyer did not always keep his pledge.—One wet, cold day, a negro man went to the Quaker's store, and the good man gave him a drink of brandy. This was brought to the notice of the temperance society, and it was decided that the offender should be severely reprimanded. The lawyer was selected to carry out this sentence, and, taking the storekeeper into the woods, he thus addressed him: "Jeemes, thee should be more circumspect!"

Dr. Condie, of Philadelphia, wished to say that he had offered the resolution in good faith, but he denied that he had made propositions to the gentleman from New York, or that the Philadelphia committee had.

Dr. Bowling of Tennessee, said that there was no question of veracity. Gentlemen on either side were correct. He had heard of misunderstanding, and of probable difficulty, and had earnestly endeavored to arrange it. He had told Dr. Reese that if he made an apology, the remonstrance would not be presented, because he had understood gentlemen from Pensylvania to say so. But he was now aware that these gentlemen did not, in any way pledge the Philadelphia county medical society.

Dr. Condie hoped that a committee would be appointed to give the subject a careful consideration.

Dr. Cox, of Maryland, after complimenting Dr. Reese as an able practitioner and an experienced editor, whose labors have been of great value to the profession and to the country, said that he did not consider the statement full and satisfactory. The offence was not an unpardonable one, but the violation of that code of ethics which is the life of the profession should be properly atoned for. The apology was good enough, but it carried as its sting the mental reservation which Dr. Reese persists in Nay, in his journal, issued simultaneously with this meeting, and circulated here, he says: "Having done right in certifying to the labors of our quondam friend McClintock, we resented the unmerited censures of our Philadelphia brethren." This completely stultifies the effect of the apology.

Dr. La Roche, of Philadelphia, explained his action and that of the Philadelphia county society in the matter.

Dr. Payne, of Virginia, Dr. Cox, and Dr. Bond made some rather sharp remarks. Dr. Davis, of Massachusetts, thought that Dr. Reese had but to admit that he had done wrong, and ask pardon without any mental reservation.

Dr. Reese said that he intended to make a satisfactory apology. Such was his earnest wish and desire, and he wished to frankly state that he had no mental reservation, neither did he attempt to conceal anything.— He made the statement that had been read without reservation and without evasion.

Dr. Condie expressed his entire satisfaction, as did numerous other gentlemen, several crossing to where Dr. Reese was sitting and shaking hands with him.

The committee of the whole then rose, and the chairman reported to the President that the committee had heard and discussed the apology of Dr. Reese, and that they considered that it was "ample, full, complete, and satisfactory."

On motion, the report of the committee was received and adopted.

The case of Dr. Bryan then came up, when it was suggested that his apology should be in writing, he expressed a willingness to make one as ample as was that of Dr. Reese.

Dr. Reese then drafted an apology, but several gentlemen insisted that he should insert the word "regret." Dr. Reese declined, saying that no gentleman would apologize for that which he did not regret, and that he would never be dictated to by any gentleman, even if the prison door stood open on his right hand, and the stake at his left.

Dr. George B. Wood (who was greeted with loud applause) stated that he had been with the side which has offered the apology, but he did not consider the apology complete, without the insertion of the word "regret."

Drs. Bonner, Clark, of New Jersey, Hard, of Illinois, Parker, of New York, and other gentlemen participated in an exciting debate on the necessity of having the word "regret" inserted.

Dr. Reese added the following sentence : "and regrets that he has incurred the displeasure of his brethren." This was not favorably received.

At length he presented the following:

"The undersigned regrets that he certified to the professional qualifications for Blockley Hospital, Philadelphia, of an expelled member of this body, and hereby offers this apology for his departure from the ethical code."

This was received with loud applause, and, on motion of Dr. White, accepted as an ample and satisfactory apology.

Dr. Bryan submitted a similar apology, which was also accepted, and the association adjourned, evidently well pleased that this question was finally disposed of.

*Third Day.*—The President, Dr. Lindsley, called the association to order at half past nine o'clock. The Secretary not having his minutes in readiness, the reading of the minutes of the day previous was dispensed with.

Dr. Grant, of New York, asked leave to present a complaint against the New York Medical College, but upon information by Dr. Edwards that a committee on ethics would be recommended by the nominating committee, he withdrew his request.

The minutes were then read. Several proposals to amend them were made, and either ruled out of order or withdrawn.

The appointment during last year of Dr. Geo. Haywood, of Boston, as a delegate to represent the American Medical Association in kindred societies in Europe, was announced by Dr. Eve.

The remainder of the proceedings of the American Medical Association will be found in the next number.

